# Positive effect of Balint group on burnout and self-efficacy of head nurses in China: a randomized controlled trial

**DOI:** 10.3389/fpsyt.2023.1265976

**Published:** 2024-01-08

**Authors:** Qu Shan, Rainer Leonhart, Xie Zhijuan, Zheng Minjie, Shi Xinxin, Bai Xinzhu, Kong Xiangyan, Kurt Fritzsche

**Affiliations:** ^1^Department of Medical Psychology, Peking University People's Hospital, Beijing, China; ^2^Institute of Psychology, University of Freiburg, Freiburg, Germany; ^3^Peking University People's Hospital, Beijing, China; ^4^Department of Nursing, Peking University People's Hospital, Beijing, China; ^5^Department of Psychosomatic Medicine and Psychotherapy, Faculty of Medicine, Medical Center University of Freiburg, Freiburg, Germany

**Keywords:** Balint group, burnout, self-efficacy, head nurses, patient relationships

## Abstract

**Background:**

Burnout is common among nurses and can lead to negative outcomes of medical care. This study aimed to explore the effectiveness of Balint groups to reduce burnout in head nurses in a Chinese hospital.

**Methods:**

This was a randomized controlled trial with a pre- and post-test. A total of 80 head nurses were randomly assigned to either a Balint group (*n* = 40) or a control group (*n* = 40). Participants participated in Balint group for a period of 3 months. Participants in both groups completed the Maslach Burnout Inventory-Human Services Survey and the General Self-Efficacy Scale at the beginning and end of the study. Balint group members also completed the Group Climate Questionnaire-Short Form.

**Results:**

In the Balint group, 33 participants attended all Balint groups, while the 40 participants in the control group had no intervention. Analysis of variance with repeated measures demonstrated a statistically significant difference on the Maslach Burnout Inventory subscale of sense of personal achievement (*F* = 9.598, *p* = 0.003) between the Balint and control groups. However, there were no significant differences between the groups on the subscales of emotional exhaustion (*F* = 0.110, *p* = 0.740) and depersonalization (*F* = 0.75, *p* = 0.387), and the General Self-Efficacy Scale (*F* = 0.709, *p* = 0.403).

**Conclusions:**

Balint groups helped reduce burnout among head nurses in terms of personal achievement.

## 1 Introduction

Head nurses perform various important and tedious tasks in clinical frontline work. They are required to take care of nursing and management work, and in particular, deal with challenging nurse-patient relationships.

However, mental overload, time shortage, communication difficulties, and perceived loss of control may lead to burnout among head nurses (1). Burnout is a prolonged stress reaction characterized by the following: emotional exhaustion—feeling overwhelmed by job demands and depleted emotional resources; depersonalization—an impersonal and detached attitude toward patients; and reduced personal achievement—a decline in feelings of work competence and achievement (2). The Maslach Burnout Inventory is used to support the management of healthcare workers in hospitals. Several studies have found high burnout levels and emotional strain among nurses, but few studies have focused on head nurses who face additional stressors given their increased responsibilities (3).

Negative emotions, frustration, and work stress may reduce nurses' confidence in their ability to complete tasks and directly damage their self-efficacy. Self-efficacy refers to the degree of an individual's confidence in completing a certain task using their skills, and indicates the expression of self-confidence in dealing with external challenges (4). Self-efficacy directly impacts nurses' work along with their physical and mental health; therefore, it is the most powerful personal resource when coping with stress, as it can reduce its negative impact. Some scholars also believe that nurses' self-efficacy can directly predict their job involvement and burnout as well as psychosomatic problems (5).

A Balint group is a working form that focuses on the professional doctor-patient relationship. Its central content focuses on the clinical professional doctor-patient relationship, helps clinical staff improve their ability to understand patients and effectively deal with their emotions and personality development, alleviates job burnout, and enhances physicians' sense of self-efficacy (6–8). Since Dr. Balint first proposed this working method in London in the 1950s, it has gradually become a compulsory course in medical education and training in some European and American countries (9). Balint group work was introduced to China by German experts more than a decade ago, and was immediately welcomed by clinical workers in various professional fields. This well-known phenomenon reflects the general distress of current clinical workers together with their hesitation and anxiety about the doctor-patient relationship. Previous studies have focused on the intervention for doctors, but studies on nurses, who are in closer contact with patients, are limited. Further, research on head nurses is even more scant, and the research period is usually short. Therefore, this study aimed to assess the effect of reducing burnout and improving self-efficacy in head nurses before and after Balint groups, and to provide theoretical support for implementing psychological interventions for frontline clinical staff.

## 2 Methods

### 2.1 Participants and recruitment

A randomized controlled trial study was conducted among head nurses in a large general hospital in Beijing, China. The inclusion criteria were: (1) head nurses in clinical departments, (2) voluntary participation, and (3) no previous participation in Balint groups. We recruited 80 head nurses through advertising. Participants were randomly assigned to either a Balint group or control group. The 40 participants in the control group were placed on the waitlist for future Balint groups but did not receive any interventions during the study period (we plan to conduct Balint groups for nurses on the waiting lists in the next year; in the next trial, we will also use the data from the waitlisted control group). The participants were all from the same hospital in Beijing, China. They were from different specialties, including internal medicine (*n* = 33), surgery (*n* = 30), gynecology and obstetrics (*n* = 4), pediatrics (*n* = 4), emergency medicine (*n* = 4), radiology (*n* = 2), anesthesiology (*n* = 2), and ophthalmology (*n* = 1). The head nurses were informed about the specific processes of the project and the potential risks and benefits. The sample size was established based on an overall difference in the outcome measure of Maslach Burnout Inventory (MBI) scores (emotional exhaustion [EE], depersonalization [DP], and personal achievement [PA]) between participants in the control and intervention groups, where a sample size of 60 was sufficient to detect a difference in MBI scores (10). Assuming a dropout rate of 15% throughout the study, the required sample size was determined as 71, with 35 participants in each group.

Before the study began, informed consent was obtained from all participants.

The study was approved by the Peking University People's Hospital Ethics Board (Registration Number 2020PHB151). The study was registered with ClinicalTrials.gov Protocol Registration (ClinicalTrials.gov Identifier: NCT05716828).

### 2.2 Measures

#### 2.2.1 Demographic variables

All participants completed a demographic questionnaire that included age, gender, clinical department, marital status, and length of medical service in mean years.

#### 2.2.2 Burnout

Burnout was measured using the Chinese version of the Maslach Burnout Inventory-Human Services Survey (MBI-HSS) (11, 12). This questionnaire comprises 22 items across three domains: Maslach Burnout Inventory-emotional exhaustion (MBI-EE) (9 items), Maslach Burnout Inventory-depersonalization (MBI-DP) (5 items), and Maslach Burnout Inventory-personal achievement (MBI-PA) (8 items). The scoring range for each item is 0 (never felt) to 6 (felt every day). The MBI-EE subscale evaluates feelings of excessive emotional stress and exhaustion due to work, which is characterized by mental, emotional, and physical exhaustion. The MBI-DP subscale measures unsympathetic and impersonal responses to patients, and is regarded as a form of depersonalization. The MBI-PA subscale assesses work-related ability and sense of achievement. The score for each subscale is calculated separately and not combined into a single total score. The scores of the scales are different: for MBI-EE and MBI-DP, higher scores represent more burnout; for MBI-PA, higher scores represent less burnout. Regarding burnout severity, an MBI-EE subscale score below 19 represents a low-level, above 26 represents a high-level, and 19–26 represents a medium-level. An MBI-DP subscale score below 6 is low, above 9 is high, and 6–9 is medium. An MBI-PA subscale score above 34 is low, below 26 is high, and 26–34 is medium (11). In a previous study, the Cronbach's alpha coefficients for the MBI-EE, MBI-DP, and MBI-PA subscales were 0.89, 0.79, and 0.87, respectively (10). The Cronbach's alpha coefficients for the MBI-EE, MBI-DP, and MBI-PA subscales in this study were 0.742, 0.803, and 0.862, respectively.

#### 2.2.3 Self-efficacy

The General Self-Efficacy Scale (GSES) uses 10 items to measure individuals' overall self-confidence in dealing with different environmental challenges or unprecedented situations. Participants are asked to rate 10 questions by choosing one of the four response options: 1 (completely incorrect), 2 (almost incorrect), 3 (relatively correct), or 4 (completely correct). A higher score indicates a higher level of general self-efficacy. The GSES is widely used in China, and the Chinese version has good reliability and validity. Schwarzer included 7,767 individuals from 13 different countries and calculated a mean score of 2.86 for this scale in the general population (13). Whereas, the internal consistency coefficient for the scale was 0.862 in previous research (6, 14, 15), its value was 0.756 in this study.

#### 2.2.4 Group climate questionnaire

The Group Climate Questionnaire-Short Form (GCQ-S) assessed the group climate using a three-dimensional construct comprising engagement, avoidance, and conflict (16). The GCQ-S engagement measures the team's self-disclosure and work orientation. The GCQ-S avoidance examines the extent to which individuals depend on other team members or leaders to create and manage team interactions while avoiding taking responsibility for their change process. The GCQ-S conflict measures hostility, including anger, distrust, and rejection. GCQ-S consists of 12 items using seven-point Likert scales ranging from not at all (0) to extremely (6). Each subscale of GCQ-S is divided into high group, low group, and medium group. The highest 27% of scores (4.38–6) are assumed as high. The lowest 27% (0–1.62) are low, and the middle score (1.63–4.37) are described as medium. A high engagement score indicates a positive working group atmosphere. A high avoidance score indicates the avoidance of personal responsibility of group work by members. A high conflict score reflects anger and tension in the group (17).

### 2.3 Procedure

The intervention program began in July 2020 and ended in January 2022. Participants in the Balint group completed Balint groups for a period of 3 months, which included two lectures and 10 small group discussion sessions held once a month for 1 h at a time (on Thursdays from 12:00 to 13:00). Participants in both groups completed the MBI-HSS and GSES questionnaires at the beginning and end of the intervention period. The Balint group also completed the GCQ-S. The GCQ-S was administered twice; the first time after the first Balint groups, and the second after the tenth Balint groups.

**Figure 1:**
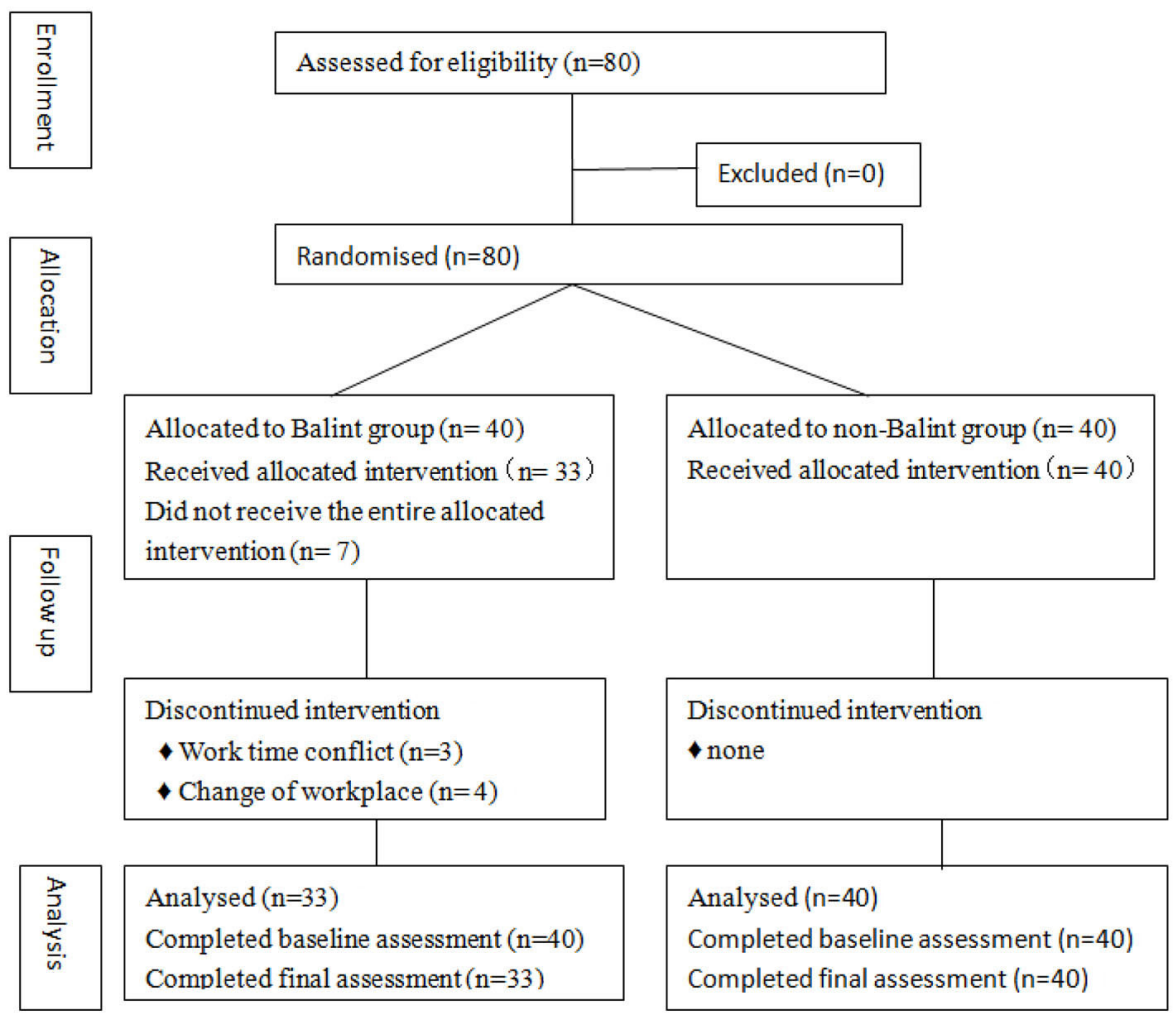
Consort flow diagram.

The study's purpose was explained to the participants and they were informed that participation was voluntary. Figure 1 shows the consort flow diagram.

### 2.4 Intervention

A Balint group intervention is a standardized model, which was introduced in London (18). Each group is a closed group including one to two group leaders and six to twelve participants, with sessions lasting 60–90 min. At the beginning of the first group session, leaders explain the basic norms and expectations for how members relate to each other during a Balint groups. The members are asked to respect these rules to ensure there is a safe talking space for all group members and leaders, allowing the group to work openly and effectively. The main expectations include: maintaining confidentiality, being respectful and non-judgmental, speaking on one's own behalf, providing an opportunity for all members to speak, avoiding offering advice or solutions, and reaching an agreement about time, place, membership, and how to work together. This is called maintaining the frame (19).

Before each meeting, all participants were invited to prepare a challenging nurse-patient encounter case. At the beginning of the meeting, possible cases were described briefly, and then the group decided which case should be taken. The presenter briefly described the selected case, and the others decided whether to choose it as the topic for that day. During the meeting, the presenter was required to: (1) spend 5–10 min describing a nurse-patient relationship that was disturbing, frustrating, confusing, or uneasy; (2) answer short factual questions from other members; (3) step back from their chair, listen to, and reflect on the discussions of other group members; (4) focus on their own feelings when listening; and (5) return to the group and analyze their reflections. Other team members were required to: (1) explore the nurse-patient relationship in the given case; (2) share what would happen to them if they were that nurse or patient; (3) introspect themselves and use their imaginations to explore the unconscious parts of the case; (4) pay attention to the differences among team members; and (5) generate new opinions and ideas on the case. The team leader was required to: (1) establish a clear agreement to create and maintain a safe group environment; (2) maintain the framework; (3) protect the care provider and other members of the group from falling into judgment and irrelevant discussion; (4) provide reasonable and timely intervention to encourage reflection, introspection, empathy, and open communication; and (5) participate in the development of the group to ensure that it follows the Balint approach to explore the nurse-patient relationship (18).

**Figure 2:**
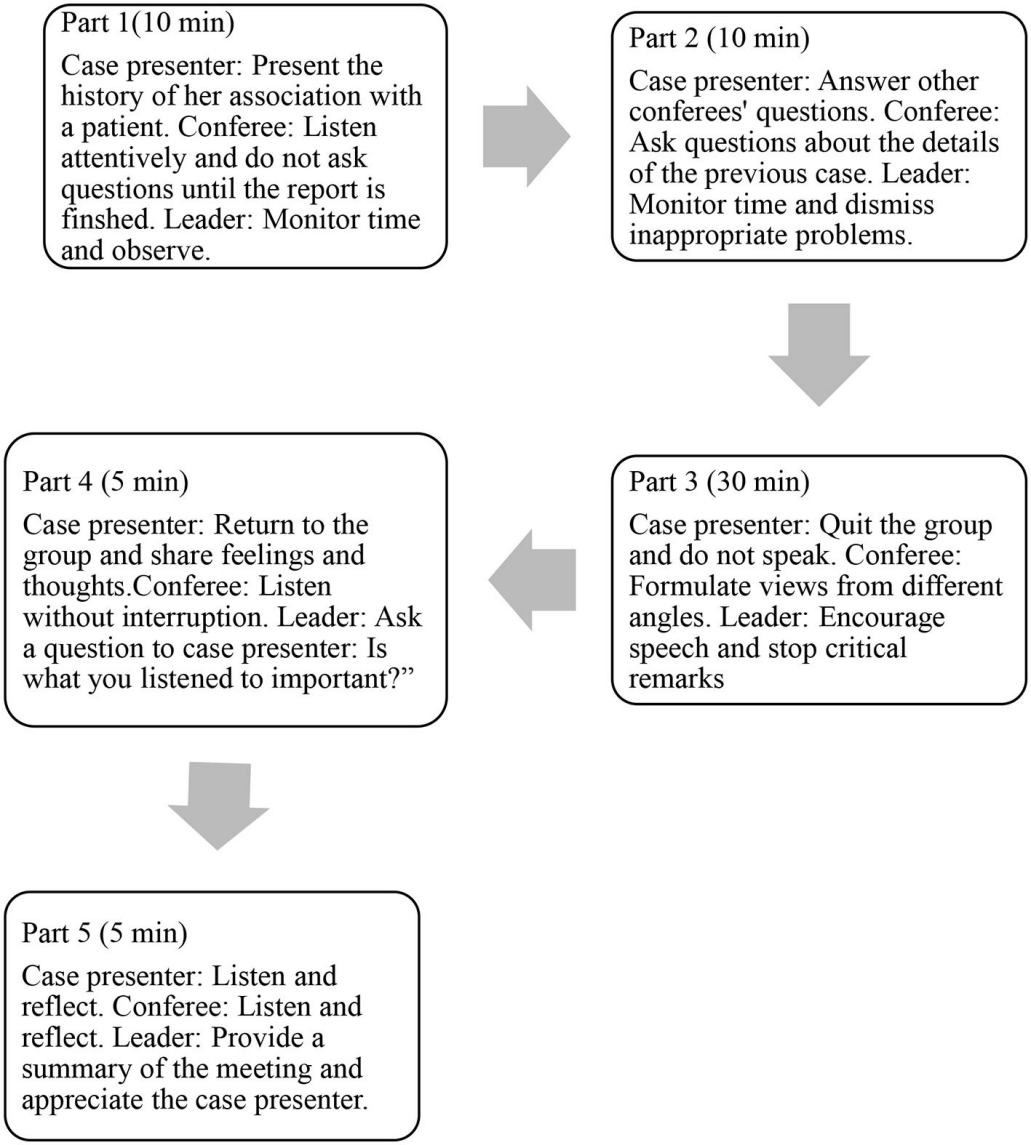
Typical process of a Balint group session.

The discussion emphasized the participants' emotions and attitudes; medical terminology was avoided, and they were not required to provide specific ways to solve problems. Participants were asked to consider their reactions, emotions, and thoughts regarding the nurse who presented the case. They were expected to consider the nurse-patient relationship from the nurse's and patient's perspectives (20). Figure 2 shows the process for the Balint group session.

Analyses were conducted using IBM SPSS Statistics version 26 (IBM, Armonk, NY, and the US) and SAS 9.4 (SAS Institute, Cary, NC). Categorical data utilized chi-square or Fisher's exact tests. At the end of the Balint groups, we used an analysis of variance for repeated measures to compare the differences between the Balint and control groups, as we expected highly correlated values. We also calculated the effect sizes (Cohen's d). According to the criteria proposed by Cohen, a value of 0.2 denotes a small effect, 0.5 a moderate effect, and 0.8 a large effect.

## 3 Results

### 3.1 Demographic characteristics

In total, 80 female participants were enrolled in this study (40 in the Balint group and 40 in the control group). In the Balint group, 33 participants completed all the interventions, and in the control group, no intervention was given to the 40 participants. In the Balint group, seven participants dropped out of the study; three stopped participating owing to conflict with work timings and four withdrew owing to changes of workplace. There were no significant differences in general demographic characteristics between the Balint and control groups (Table 1).

**Table 1:** Demographic characteristics of the participants.

**Variable**	**Balint group (*n =* 40)**	**Control group (*n =* 40)**	**df**	***t*-test/X^2^**	***p*-value**
Age [mean years (SD)]	47.3 (6.7)	46.4 (6.9)	78	0.493	0.700
Age (range)	(34–59)	(35–57)			
Marital status			1	2.000	0.157
Married	35	37			
Single	5	3			
Length of medical service [mean years (SD)]	27.4 (6.7)	27.0 (6.5)	78	0.493	0.799
Length of education [mean years (SD)]	15.6 (0.6)	15.3 (0.8)	78	1.709	0.091

### 3.2 Comparison of burnout and GSES scores between the Balint and control groups

After the Balint groups, a statistically significant difference was found in the score on the MBI-PA subscale (95% CI: 8.28, 11.12, df = 1, f = 9.598, *p* = 0.003) between the Balint and control group. After the Balint groups, the mean score of the MBI-PA subscale in the Balint group was higher than it before Balint groups. This meant that this aspect of burnout had decreased. By contrast, in the second measurement, the mean score of the MBI-PA subscale in the control group was lower than it was at the first measurement (start of the Balint groups). The negative change in the control group indicated perhaps the aggravation of burnout. However, there was no statistical difference between the groups on the MBI-EE (df = 1, F = 0.110, *p* = 0.740), and MBI-DP subscales (df = 1, *F* = 0.757, *p* = 0.387) or the GSES (df = 1, *F* = 0.709, *p* = 0.403). There was no significant difference in burnout severity between the Balint and control groups before Balint groups: the MBI-EE subscale was at a high level, and the MBI-DP and MBI-PA were both at the medium level (Table 2).

**Table 2:** Comparison of the Balint and control groups' scores on the MBI and GSES.

	**Balint group (n =** **33)**		**Control group (n =** **40)**		**Difference between groups after the Balint groups (95% CI)**	**Cohen's d**	**df**	**F**	***p***
	**Before (mean** ±**SD)**	**After (mean** ±**SD)**	**Before (mean** ±**SD)**	**After (mean** ±**SD)**					
MBI-EE	32.42 ± 11.71	28.30 ± 7.73	31.85 ± 11.33	27.65 ± 3.64	0.49 (−1.53, 2.51)	0.002	1	0.110	0.740
MBI-DP	8.88 ± 7.14	6.18 ± 3.74	8.87 ± 6.74	7.97 ± 2.45	−1.87 (−3.22, 0.51)	0.011	1	0.757	0.387
MBI-PA	26.91 ± 10.26	31.15 ± 4.60	27.30 ± 9.74	21.70 ± 4.72	9.70 (8.28, 11.12)	0.119	1	9.598	0.003
GSES	1.97 ± 0.73	2.24 ± 0.23	2.02 ± 0.16	2.29 ± 0.18	−0.05 (−0.15, 0.04)	0.010	1	0.709	0.403

MBI-EE, Maslach Burnout Inventory (MBI) scores-emotional exhaustion; MBI-DP, Maslach Burnout Inventory (MBI) scores-depersonalization; MBI-PA, Maslach Burnout Inventory (MBI) scores-personal achievement; GSES, General Self-Efficacy Scale.

### 3.3 Comparison of group climate before and after Balint groups

Before the Balint groups, the mean score on the engagement subscale (4.34) was at a high level, the mean score on the conflict subscale (1.31) was at a low level and the mean score on the avoidance (2.82) was at a medium level, which indicated that the group climate was positive and friendly from the start. Paired *t*-test results demonstrated no statistically significant differences regarding group climate before and after the Balint groups. The scores for engagement (*t* = 0.336, *p* = 0.739) and avoidance (*t* = 0.218, *p* = 0.829) decreased after the Balint groups but the difference was not statistically significant. The score for conflict (*t* = 0.066, *p* = 0.948) increased after the Balint groups, but not significantly (Table 3).

**Table 3:** Comparison of group climate before and after Balint groups in Balint group.

	**Before Balint groups**		**After Balint groups**		**df**	**t**	***p*-value**
**Variable**	**Mean**	**SD**	**Mean**	**SD**			
Engagement	4.34	1.00	4.25	1.02	32	0.336	0.739
Conflict	1.31	1.54	1.33	1.52	32	0.066	0.948
Avoidance	2.82	1.24	2.76	1.18	32	0.218	0.829

## 4 Discussion

Balint groups helps clinical staff better understand patients, detect and deal with their emotions, develop their personality, improve their self-efficacy, and alleviate symptoms of job burnout (7). This study explored the effect of Balint groups on improvements in nurses' self-efficacy and the alleviation of job burnout.

The participants included in this study (Balint group and control group) were at moderate to severe level of burnout before the Balint groups, which meant they were under higher levels of stress.

Seven participants in the intervention group dropped out of the study. Our Balint groups were conducted in the main hospital district, and some participants were assigned work in other hospital districts during the study. Therefore, they did not have time to participate in the Balint groups and dropped out of the study because of changes in place and time of work; the dropouts were not attributable to the Balint group intervention. This is a typical phenomenon in a Chinese hospital. Staff members in the control group also experienced changes in their working areas, but this did not affect their participation in the study as they were not required to attend sessions. A previous study reported a similar situation of declining participant attendance (21).

The current results showed that head nurses' average GSES scores improved after the Balint group intervention, but the difference was not statistically significant. Previous studies reported similar findings (8). In Rabin's research program, which found significant improvement in self-efficacy, Balint groups lasted for more than a year (22). The formation and development of self-efficacy is usually a relatively long process. Our research suggests that improving self-efficacy through Balint groups may require a longer intervention period, which also provides scope for future research.

### 4.1 Burnout

We only found one significant effect among the several scales analyzed. Presumably, the negative change observed in the control group suggests that no significant effect was observed in the scales. The only exception was the MBI-PA subscale.

This finding is consistent with previous studies (23). In Stojanovic-Tasic et al.'s study, 21.4% and 7.1% of control and Balint group participants, respectively, had a statistically significant improvement in perception of PA (24). Bar-Sela et al. reported similar results and found that Balint groups improved the communication abilities of residents and contributed to their feelings of self-achievement as doctors (8). Compared with other intervention methods that pay more attention to emotional regulation, Balint groups can inspire participants to identify different perspectives to understand and manage difficult working relationships and challenging patient communication (25, 26).

Our study had a special feature: the negative changes in Personal achievement in the control group were particularly noticeable. The participants in the control group were full of expectations when they found out about Balint groups, but when they were randomly assigned to the control group, they were perhaps disappointed, which may have lowered their sense of achievement.

The reason the Balint groups effect did not show up more could have been that the subjects of this study were at a serious burnout level and high pressure. Ten one-hour sessions were far too few to bring about a significant change in their experience of burnout.

Huang et al. found that compared to the control group, EE and DP improved in the Balint group; their Balint groups lasted 1 year (10). Furthermore, Popa-Velea et al. also found that EE and DP in the Balint group improved. The study was conducted over 2 years This suggests that improvements in EE and DP may require a longer course of Balint intervention (27).

Group climate is an important measure of the group therapy effect (28, 29). The GCQ-S can be used as a predictor of the long-term efficacy of group therapy (30). In our study, there was no significant difference in the GCQ-S before and after the Balint groups. The reason was that at the beginning of Balint groups, the group atmosphere was friendly and positive with high participation and little conflict. This is consistent with previous research. Maurizio et al.'s findings were similar: after Balint groups for nurses and physicians, the subscale scores for the GCQ-S did not change (31).

Another reason was that our research intervention was not long enough to find changes in various factors of the group atmosphere. A study examined the development of group climate in short-term (20 sessions) and long-term (80 sessions) psychodynamic group psychotherapy, and found that from session 10 to session 18, GCQ-S avoidance and conflict subscales decreased in the short-term groups while long-term groups displayed the opposite pattern (29). Compared with this, our study was relatively short, and not adequate to discover changes in various factors in the group atmosphere.

Generally, the results showed that Balint groups reduced some parts of head nurses' burnout. The mechanism of the Balint group's effectiveness is analyzed as follows: conventioneers provided varying feelings and opinions on the cases. Participants were required to observe and reflect on their behaviors, difficulties, and setbacks, analyze the causes of problems, gain an understanding of cases from different angles, improve their self-awareness ability, and be able to identify, judge, and understand their complex emotions as well as those of the patients in many aspects. While finding and solving predicaments, nurses learned to affirm themselves.

### 4.2 Limitations

This study had a small sample size and all participants were from a single hospital. They were therefore not representative of the entire head nurse population. The Balint group intervention period was short and we intend to address these deficiencies in future studies. Regarding a desirable sample size, we did not consider that there were a total of four different variables and two questionnaire instruments. As we did not make adjustments for alpha errors, the results must be seen as preminilary.

## 5 Conclusion

A 3-month Balint group intervention helped reduce some part of burnout among head nurses in a Chinese hospital. No significant improvement was found in nurses' self-efficacy. In the Balint group, there was no significant change in the group climate before and after the intervention.

The research results provide directions for better and more efficient medical education programs using Balint activities. Balint groups can guide nurses to become more patient-centered by reducing burnout levels.

## Data availability statement

The original contributions presented in the study are included in the article/Supplementary material, further inquiries can be directed to the corresponding author.

## Ethics statement

The studies involving humans were approved by Peking University People's Hospital Ethics Board. The studies were conducted in accordance with the local legislation and institutional requirements. The participants provided their written informed consent to participate in this study.

## Author contributions

QS: Writing—original draft, Writing—review & editing. RL: Data curation, Writing—review & editing. XZ: Investigation, Writing—review & editing. ZM: Data curation, Software, Writing—review & editing. SX: Conceptualization, Investigation, Writing—review & editing. BX: Investigation, Software, Writing—review & editing. KX: Resources, Supervision, Writing—review & editing. KF: Methodology, Writing—review & editing.

## References

[1] Nwanya M Rowberry D. The importance of understanding burnout: an oncology nurse perspective. Br J Nurs. (2021) 30:S8–14. 10.12968/bjon.2021.30.10.S8 34037439

[2] Brusaferro S Agnoletto AP Gubian F Balestrieri M. Use of the Maslach Burnout Inventory to support health care workers management in hospitals. J Prev Med Hyg. (2000) 41:18–23.

[3] Kelly LA Lefton C Fischer SA. Nurse leader burnout, satisfaction, and work-life balance. J Nurs Adm. (2019) 49:404–10. 10.1097/NNA.0000000000000784 31425307

[4] Bandura A Adams NE Beyer J. Cognitive processes mediating behavioral change. J Pers Soc Psychol. (1977) 35:125–39. 10.1037/0022-3514.35.3.125 15093

[5] Molero Jurado MDM Pérez-Fuentes MDC Oropesa Ruiz NF Simón Márquez MDM Gázquez Linares JJ. Self-efficacy and emotional intelligence as predictors of perceived stress in nursing professionals. Medicina. (2019) 55:237. 10.3390/medicina55060237 31159453 PMC6630601

[6] Yang C Zhou B Wang J Pan S. The effect of a short-term Balint group on the communication ability and self-efficacy of pre-examination and triage nurses during COVID-19. J Clin Nurs. (2021) 30:93–100. 10.1111/jocn.15489 32920947

[7] Otten H. The Theory and Practice of Balint Group Work: Analyzing Professional Relationships. London: Routledge (2017).

[8] Bar-Sela G Lulav-Grinwald D Mitnik I. “Balint group” meetings for oncology residents as a tool to improve therapeutic communication skills and reduce burnout level. J Cancer Educ. (2012) 27:786–9. 10.1007/s13187-012-0407-3 22923383

[9] Oliver S. How doctors learn in a Balint group. Fam Pract. (1989) 6:108–13. 10.1093/fampra/6.2.108 2744294

[10] Huang L Harsh J Cui H Wu J Thai J Zhang X. A randomized controlled trial of Balint groups to prevent burnout among residents in China. Front Psychiatry. (2020) 10:957. 10.3389/fpsyt.2019.00957 32116808 PMC7026367

[11] Maslach C Jackson SE Leiter MP. Maslach burnout inventory. Zalaquett CP Wood RJ, Editors. In: *Evaluating Stress: A Book of Resources*. 3rd, ed. Lanham, ML: The Scarecrow Press (1997).

[12] Chen R Sun C Chen JJ Jen HJ Kang XL Kao CC. large-scale survey on trauma, burnout, and posttraumatic growth among nurses during the COVID-19 pandemic. Int J Ment Health Nurs. (2021) 30:102–16. 10.1111/inm.12796 33107677 PMC7894338

[13] Schwarzer R. Optimistic self-beliefs: Assessment of general perceived self-efficacy in thirteen cultures. World Psychol. (1997) 3:177–90.

[14] Wang N Wang S Qian H Ruan Y Amico KR Vermund SH. Negative associations between general self-efficacy and anxiety/depression among newly HIV-diagnosed men who have sex with men in Beijing, China. AIDS Care. (2019) 31:629–35. 10.1080/09540121.2018.1549721 30466302 PMC7942229

[15] Wang ZY Liu L Shi M Wang L. Exploring correlations between positive psychological resources and symptoms of psychological distress among hematological cancer patients: a cross-sectional study. Psychol Health Med. (2016) 21:571–82. 10.1080/13548506.2015.1127396 26708250

[16] Dies RR MacKenzie KR. Advances in Group Psychotherapy: Integrating Research and Practice. New York, NY: International Universities Press (1983).

[17] Gold PB Kivlighan Jr DM Patton MJ. Accounting for session-level dependencies in longitudinal associations of group climate and therapeutic factors in interpersonally focused counselor-training groups. Group Dyn Theory Res Pract. (2013) 2:81–94. 10.1037/a0031773

[18] Fritzsche K Scheib P Ko N Wirsching M Kuhnert A Hick J. Results of a psychosomatic training program in China, Vietnam and Laos: successful cross-cultural transfer of a postgraduate training program for medical doctors. Biopsychosoc Med. (2012) 6:1–4. 10.1186/1751-0759-6-17 22929520 PMC3546304

[19] Fritzsche K Shi L Wei J. Self-awareness in the training of health care professionals: validation of a group self-awareness questionnaire among Balint groups in China. Res Sq. (2020) 1–20. 10.21203/rs.3.rs-33641/v1

[20] Ghetti C Chang J Gosman G. Burnout, psychological skills, and empathy: Balint training in obstetrics and gynecology residents. J Grad Med Educ. (2009) 1:231–5. 10.4300/JGME-D-09-00049.1 21975984 PMC2931236

[21] Koppe H van de Mortel TF Ahern CM. How effective and acceptable is Web 20 Balint group participation for general practitioners and general practitioner registrars in regional Australia? A pilot study. Aust J Rural Health. (2016) 24:16–22. 10.1111/ajr.12212 26114400 PMC4755195

[22] Rabin S Herz M Stern M Vaserfirer I Belakovsky S Mark M. Improving the professional self-efficacy cognitions of immigrant doctors with Balint groups. Isr J Psychiatry Relat Sci. (1996) 33:253–9. 9066209

[23] Zhang XJ Song Y Jiang T Ding N Shi TY. Interventions to reduce burnout of physicians and nurses: an overview of systematic reviews and meta-analyses. Medicine. (2020) 99:e20992. 10.1097/MD.0000000000020992 32590814 PMC7328917

[24] Stojanovic-Tasic M Latas M Milosevic N Aritonovic PJ Ljusic D Sapic R. Is Balint training associated with the reduced burnout among primary health care doctors? Libyan J Med. (2018) 13:1440123. 10.1080/19932820.2018.1440123 29493438 PMC5844034

[25] Kjeldmand D Holmström I Rosenqvist U. Balint training makes GPs thrive better in their job. Patient Educ Couns. (2004) 55:230–5. 10.1016/j.pec.2003.09.009 15530759

[26] Torppa MA Makkonen E Mårtenson C Pitkälä KH. A qualitative analysis of student Balint groups in medical education: contexts and triggers of case presentations and discussion themes. Patient Educ Couns. (2008) 72:5–11. 10.1016/j.pec.2008.01.012 18295432

[27] Popa-Velea O Trutescu CI Diaconescu LV. The impact of Balint work on alexithymia, perceived stress, perceived social support and burnout among physicians working in palliative care: a longitudinal study. Int J Occup Med Environ Health. (2019) 32:53–63. 10.13075/ijomeh.1896.01302 30785127

[28] Manne SL Kashy D Siegel SD Heckman CJ. Group therapy processes and treatment outcomes in 2 couple-focused group interventions for breast cancer patients. Psychooncology. (2017) 26:2175–85. 10.1002/pon.4323 27885746 PMC5548627

[29] Bakali JV Wilberg T Klungsøyr O Lorentzen S. Development of group climate in short- and long-term psychodynamic group psychotherapy. Int J Group Psychother. (2013) 63:366–93. 10.1521/ijgp.2013.63.3.366 23734920

[30] Bonsaksen T Borge F Hoffart A. Group climate as predictor of short- and long-term outcome in group therapy for social phobia. Int J Group Psychother. (2013) 63:394–417. 10.1521/ijgp.2013.63.3.394 23734921

[31] Abeni MS Magni M Conte M Mangiacavalli S Pochintesta L Vicenzi G. Psychological care of caregivers, nurses and physicians: a study of a new approach. Cancer Med. (2014) 3:101–10. 10.1002/cam4.163 24402889 PMC3930394

